# Is eating behavior manipulated by the gastrointestinal microbiota? Evolutionary pressures and potential mechanisms

**DOI:** 10.1002/bies.201400071

**Published:** 2014-08-08

**Authors:** Joe Alcock, Carlo C Maley, C Athena Aktipis

**Affiliations:** 1) Department of Emergency Medicine, University of New MexicoAlbuquerque, NM, USA; 2) Center for Evolution and Cancer, Helen Diller Family Comprehensive Cancer CenterSan Francisco, CA, USA; 3) Department of Surgery, University of California San FranciscoSan Francisco, CA, USA; 4) Wissenschaftskolleg zu Berlin, (Institute for Advanced Study Berlin)Berlin, Germany; 5) Department of Psychology, Arizona State UniversityTempe, AZ, USA

**Keywords:** cravings, evolutionary conflict, host manipulation, microbiome, microbiota, obesity

## Abstract

Microbes in the gastrointestinal tract are under selective pressure to manipulate host eating behavior to increase their fitness, sometimes at the expense of host fitness. Microbes may do this through two potential strategies: (i) generating cravings for foods that they specialize on or foods that suppress their competitors, or (ii) inducing dysphoria until we eat foods that enhance their fitness. We review several potential mechanisms for microbial control over eating behavior including microbial influence on reward and satiety pathways, production of toxins that alter mood, changes to receptors including taste receptors, and hijacking of the vagus nerve, the neural axis between the gut and the brain. We also review the evidence for alternative explanations for cravings and unhealthy eating behavior. Because microbiota are easily manipulatable by prebiotics, probiotics, antibiotics, fecal transplants, and dietary changes, altering our microbiota offers a tractable approach to otherwise intractable problems of obesity and unhealthy eating.

## Introduction: Evolutionary conflict between host and microbes leads to host manipulation

The struggle to resist cravings for foods that are high in sugar and fat is part of daily life for many people. Unhealthy eating is a major contributor to health problems including obesity 1 as well as sleep apnea, diabetes, heart disease, and cancer 2–4. Despite negative effects on health and survival, unhealthy eating patterns are often difficult to change. The resistance to change is frequently framed as a matter of “self-control,” and it has been suggested that multiple “selves” or cognitive modules exist 5 each vying for control over our eating behavior. Here, we suggest another possibility: that evolutionary conflict between host and microbes in the gut leads microbes to divergent interests over host eating behavior. Gut microbes may manipulate host eating behavior in ways that promote their fitness at the expense of host fitness. Others have hypothesized that microbes may be affecting our eating behavior 6–8, though not in the context of competing fitness interests and evolutionary conflict.

Conflict over resource acquisition and resource allocation can occur as a result of conflict between different genetic interests within an organism. For example, genetic conflict between maternal and paternal genes is hypothesized to play a role in the unusual eating behavior that characterizes the childhood genetic diseases Beckwith–Wiedemann syndrome and Prader–Willi syndrome. These syndromes are characterized by altered appetite and differences in infant suckling that can result from overexpression of genes of paternal or maternal origin, respectively 9,10. In parent-of-origin genetic conflict, paternally imprinted genes are thought to drive increased demands for extracting resources from the mother, and maternally imprinted genes tend to resist these effects. Metagenomic conflict between host and microbiome can be considered an extension of this genetic conflict framework, but one that includes other genomes (i.e., microbes in the gut) with genes that affect the physiology and behavior of a host organism, potentially altering host eating behavior in ways that benefit microbe fitness.

Microbial genes outnumber human genes by 100 to 1 in the intestinal microbiome, leading some to propose that it is a “microbial organ” that performs important functions for the host, such as nutrient harvesting and immune development 11. However, as with any complex and intimate interaction, there is a mixture of common and divergent interests with opportunities for mutual benefit 11 and manipulation 12. Fitness interests of gut microbes are also often not aligned, because members of the microbiota compete with one another over habitat and nutrients. This means that highly diverse populations of gut microbes may be more likely to expend energy and resources in competition, compared to a less diverse microbial population. A less diverse microbial population is likely to have species within it that have large population sizes and more resources available for host manipulation. Moreover, the larger a particular microbial population is, the more power it would have to manipulate the host through higher levels of factor production or other strategies (see below) and large scale coordination of these activities (e.g., through quorum sensing). Therefore, we hypothesize that lower diversity in gut microbiome should be associated with more unhealthy eating behavior and greater obesity (i.e., decreased host fitness).

## Evidence indicates many potential mechanisms of manipulation

### There is a selective influence of diet on microbiota

Individual members of the microbiota, and consortia of those microbes, have been shown to be highly dependent on the nutrient composition of the diet. *Prevotella* grows best on carbohydrates; dietary fiber provides a competitive advantage to *Bifidobacteria* 13, and *Bacteroidetes* has a substrate preference for certain fats 14. Some specialist microbes, e.g. mucin degrading bacteria such as *Akkermansia mucinophila*, thrive on secreted carbohydrates provided by host cells. Other butyrate producing microbes, e.g. *Roseburia* spp., fare better when they are delivered polysaccharide growth substrates in the diet. Specialist microbes that digest seaweed have been isolated from humans in Japan 15. African children raised on sorghum have unique microbes that digest cellulose 16. Many other examples exist 17. Even microbes with a generalist strategy tend to do better on some combinations of nutrients than others, and competition will determine which microbes survive 18,19.

### Microbes can manipulate host behavior

There is circumstantial evidence for a connection between cravings and the composition of gut microbiota. Individuals who are “chocolate desiring” have different microbial metabolites in their urine than “chocolate indifferent” individuals, despite eating identical diets 20. There is also evidence for effects of microbes on mood. A double-blind, randomized, placebo controlled trial found that mood was significantly improved by drinking probiotic *Lactobacillus casei* in participants whose mood was initially in the lowest tertile 21.

**Figure 1:**
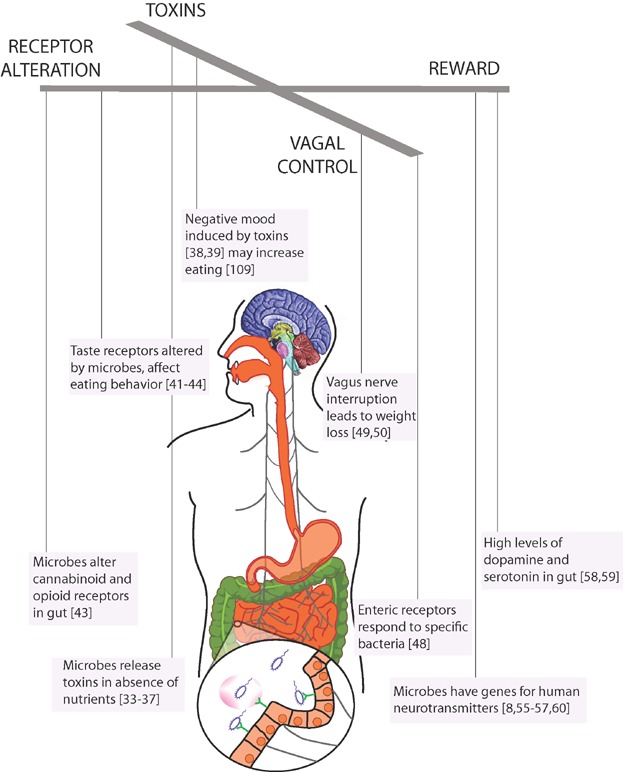
Like microscopic puppetmasters, microbes may control the eating behavior of hosts through a number of potential mechanisms including microbial manipulation of reward pathways, production of toxins that alter mood (shown in pink, diffusing from a microbe), changes to receptors including taste receptors, and hijacking of neurotransmission via the vagus nerve (gray), which is the main neural axis between the gut and the brain.

There are many other examples of microbes affecting their hosts' mood and behavior, mostly from animal studies (Fig. 1). Butyrate, a short chain fatty acid largely produced by the microbiota, has been shown to have profound central nervous system effects on mood and behavior in mice 22. Microbiota transfer to germ free mice leads to timid behavior when fed feces from mice with anxiety-like behavior. When germ-free mice from an anxious strain were fed with a fecal pellet from a control mouse, the inoculated mice exhibited behavior that was more exploratory, and more like their fecal donors 23. In addition, a probiotic formulation with *Lactobacillus helveticus R0052* and *Bifidobacterium longum R0175* alleviated psychological distress 24. This effect can be altered by diet and inflammation 25. If one feeds *Lactobacillus rhamnosus* (*JB-1*) to mice, not only does it reduce their stress-induced corticosterone hormone levels, but it also makes them more dogged: *L. rhamnosus* (*JB-1*) fed mice swim longer than the control fed mice when put in a glass cylinder filled with 15 cm of water and no means of escape 26. This effect disappeared when the experimenters severed the vagus nerve, suggesting a role for the vagus nerve in microbial manipulation of host behavior. In contrast, severing the vagus nerve had no effect on swimming behavior of control mice that were not fed *L. rhamnosus* (*JB-1*) 26. In a widely cited example of microbes affecting behavior, *Toxoplasma gondii* suppresses rats' normal fear of cat smells, often to the detriment of the rats, but to the benefit of the microbes that are ingested into their new feline host. *T. gondii* infected rats are reported to become sexually aroused by cat urine 27, a propensity that promotes transmission of *T. gondii* at the expense of the fitness of the rat.

### Microbes can induce dysphoria that changes feeding behavior

Although certain *Lactobacillus* appear to reduce anxiety, colonization of the gut with the pathogen *Campylobacter jejuni* increased anxiety-like behavior in mice 28, raising the possibility that microbe-induced dysphoria might also affect human behavior. Recent studies have linked the inconsolable crying of infant colic with changes in gut microbiota including reduced overall diversity, increased density of Proteobacteria and decreased numbers of Bacteroidetes compared to controls 29. Colic has been reported to result in increased energy delivery to infants, sometimes resulting in accelerated weight gain 30. If infant crying has a signaling function that increases parental attention and feeding 31,32, colic may increase the resource delivery to the gut and hence microbial access to nutrients.

One potential mechanism by which dysphoria can influence eating involves bacterial virulence gene expression and host pain perception. This mode of manipulation is plausible because production of virulence toxins often is triggered by a low concentration of growth-limiting nutrients. Detection of simple sugars and other nutrients regulates virulence and growth for a variety of human-associated microbes 33–37. These commensals directly injure the intestinal epithelium when certain nutrients are absent, raising the possibility that microbes manipulate behaviors through pain signaling. In accord with this hypothesis, bacterial virulence proteins have been shown to activate pain receptors 38. Moreover, pain perception (nociception) requires the presence of an intestinal microbiota in mice 39 and fasting has been shown to increase nociception in rodents by a vagal nerve mechanism 40.

### Microbes modulate host receptor expression

One route to manipulation of host eating behavior is to alter the preferences of hosts through changing receptor expression. One study found that germ-free mice had altered taste receptors for fat on their tongues and in their intestines compared to mice with a normal microbiome 41. In another experiment, germ free mice preferred more sweets and had greater numbers of sweet taste receptors in the gastrointestinal tract compared to normal mice 42. In addition, *L. acidophilus* NCFM, administered orally as a probiotic, increased intestinal expression of cannabinoid and opioid receptors in mouse and rat intestines, and had similar effects in human epithelial cell culture 43. These results suggest that microbes could influence food preferences by altering receptor expression or transduction. Changes in taste receptor expression and activity have been reported after gastric bypass surgery, a procedure that also changes gut microbiota and alters satiety and food preferences (reviewed in 44).

### Microbes can influence hosts through neural mechanisms

Gut microbes may manipulate eating behavior by hijacking their host's nervous system. Evidence shows that microbes can have dramatic effects on behavior through the microbiome-gut-brain axis 6,45,46. The vagus nerve is a central actor in this communication axis, connecting the 100 million neurons of the enteric nervous system in the gut 47 to the base of the brain at the medulla. Enteric nerves have receptors that react to the presence of particular bacteria 48 and to bacterial metabolites such as short-chain fatty acids.

Evidence suggests that the vagus nerve regulates eating behavior and body weight. For example, blockade or transection of the vagus nerve has been reported to cause drastic weight loss 49,50. On the other hand, vagus nerve activity appears to drive excessive eating behavior in satiated rats when they are stimulated by norepinephrine 51. These results suggest that gut microbes that produce adrenergic neurochemicals (discussed below) may contribute to overeating via mechanisms involving vagal nerve activity.

Together these results suggest that microbes have opportunities to manipulate vagus nerve traffic in order to control host eating. Intriguingly, many practices that are known to enhance parasympathetic outflow from the vagus nerve, e.g. exercise, yoga, and meditation, are also thought to strengthen willpower 52 and improve accuracy of food intake relative to energy expenditure 53. However, increased vagus activity is not always associated with health. One study linked parasympathetic vagus activity with weight loss in patients with anorexia nervosa 54, suggesting that vagus nerve signaling is important in regulating body weight, and sometimes can lead to pathological anorexia.

### Microbes can influence hosts through hormones

Microbes produce a variety of neurochemicals that are exact analogs of mammalian hormones involved in mood and behavior 8,55–57. More than 50% of the dopamine and the vast majority of the body's serotonin have an intestinal source 58,59. Many transient and persistent inhabitants of the gut, including *Escherichia coli*, 8,55,56 *Bacillus cereus*, *B. mycoides*, *B. subtilis*, *Proteus vulgaris*, *Serratia marcescens*, and *Staphylococcus aureus* 60 have been shown to manufacture dopamine. Concentrations of dopamine in culture of these bacteria were reported to be 10–100 times higher than the typical concentration in human blood 60. *B. subtilis* appears to secrete both dopamine and norepinephrine into their environment, where it interacts with mammalian cells. Transplant of the microbiome from a male to an immature female mouse significantly and stably increases testosterone levels in the recipient 61. In turn, host enzymes are known to degrade neurotransmitters of bacterial origin. For instance, mammals use monoamine oxidase to silence exogenous signaling molecules, among other functions 62,63. This may be evidence for selection on hosts to counteract microbial interference with host signaling.

Certain probiotic strains alter the plasma levels of other neurochemicals. *B. infantis 35624* raises tryptophan levels in plasma, a precursor to serotonin 64. The lactic acid producing bacteria found in breast milk and yogurt also produce the neurochemicals histamine 65 and GABA 66. GABA activates the same neuroreceptors that are targeted by anti-anxiety drugs such as valium and other benzodiazepines.

Appetite-regulating hormones are another potential avenue for manipulation of mammalian eating behavior. In mice, treatment with VSL#3, a dietary supplement consisting of a mixture of *Lactobacillus* strains, reduced hunger-inducing hormones AgRP (agouti related protein) and neuropeptide Y in the hypothalamus 67. Germ-free mice were also shown to have lower levels of leptin, cholecystokinin, and other satiety peptides 41, hormones that control hunger and food intake partly by affecting vagus nerve signaling. Numerous commensal and pathogenic bacteria manufacture peptides that are strikingly similar to leptin, ghrelin, peptide YY, neuropeptide Y, mammalian hormones that regulate satiety and hunger 68. Moreover, humans and other mammals produce antibodies directed against these microbial peptides, a phenomenon that could have evolved as a mammalian counter-adaptation to microbial manipulation. Anti-hormone antibody production may be important in maintaining the fidelity of host signaling systems. However, these antibodies also act as auto-antibodies against mammalian hormones 68. This autoimmune response implies that microbes have the capacity to manipulate human eating behavior (i) directly with peptide mimics of satiety regulating hormones, or (ii) indirectly by stimulating production of auto-antibodies that interfere with appetite regulation. The antibody response to microbial analogs of human hormones supports the hypothesis that conflict between host and microbiota influences the regulation of eating behavior.

### Mucin foraging bacteria control their nutrient supply

Several commensal bacteria are known to induce their hosts to provide their preferred nutrients through direct manipulation of intestinal cells. For example, *Bacteroides thetaiotaomicron* is found on host mucus, where it scavenges N-glycated oligosaccharides secreted by goblet cells in the gut. *B. thetaiotaomicron* induces its mammalian host to increase goblet cell secretion of glycated carbohydrates 69,70. Investigators have shown that another mucin-feeding species, *A. muciniphila*, also increases the number of mucus producing goblet cells when inoculated in to mice 71. On the other hand *Faecalibacterium prausnitzii*, a non-mucus-degrading bacterium that is co-associated with *B. thetaiotaomicron*, inhibits mucus production by goblet cells 70. These species provide a proof of principle that gut bacteria can control their nutrient delivery, involving a mechanism that is energetically costly for the host 72.

### Intestinal microbiota can affect obesity

Evolutionary conflict between the gut microbiome and host may be an important contributor to the epidemic of obesity. In a landmark paper, Backhed and colleagues showed that mice genetically predisposed to obesity remained lean when they were raised without microbiota 73. These germfree mice were transformed into obese mice when fed a fecal pellet from a conventionally raised obese mouse 74. Inoculation of germ-free mice with microbiota from an obese human produced similar results 75. Mice lacking the toll-like receptor TLR5 became obese and developed altered gut microbiota, hyperphagia, insulin resistance, and pro-inflammatory gene expression 76. Fecal pellets from these TLR5 knockout mice, when fed to wild type mice, induced the same phenotype. The gut microbes of obese humans are less diverse than the microbiota of their lean twins 77, consistent with the hypothesis that lower diversity may affect eating behavior and satiety.

### Probiotics are associated with weight loss

The addition of probiotics (i.e. purportedly beneficial ingestible microbes) to the diet tends to decrease food intake, consistent with the hypothesis that greater gut diversity may limit microbial control over eating behavior. Some *Lactobacillus* probiotics have been reported to reduce fat mass and improve insulin sensitivity and glucose tolerance, although these effects are not universally reported for all *Lactobacillus* species 78,79. A recent study demonstrated that the probiotic VSL#3 caused mice to decrease food intake 67. Similarly, the probiotic *Bifidobacterium breve* inhibited weight gain in mice given a high fat diet in a dose-dependent manner 80. Several studies suggest a role for probiotics in weight loss in humans. In one trial, a probiotic yogurt produced weight loss that was not due to change in energy intake or exercise 81. Similarly, yogurt was the food most associated with reduced weight gain in a study that monitored the diet and health of 120,000 nurses for over 12–20 years 82. Further, a randomized, placebo-controlled trial found that probiotic treatment in pregnancy, using *L. rhamnosus* GG and *Bifidobacterium lactis* along with dietary counseling, reduced abdominal fat at 6 months post-partum 83. Together these results demonstrate that probiotics can lead to weight loss and regulate energy balance.

## Predictions and experiments

### Changing the microbiota composition will change eating behavior

Prebiotics (i.e. non-digestible compounds that stimulate growth of beneficial microbes), probiotics, antibiotics, fecal transplant, and diet changes are potential strategies to alter the microbiota. In addition to the proposal that microbiota transplantation should result in adoptive transfer of food preferences 84, we further predict that inoculation of an experimental animal with a microbe that has a specialized nutrient requirement, such as seaweed 15,85, would lead to preference for that novel food.

### A consistent diet will select for microbial specialists and lead to preference for those foods

Raising an experimental animal on a simple diet with few types of foods, should select for microbes that specialize on those foods. Our hypothesis as to the microbial origin of food preferences predicts that these microbes will influence their host to choose the foods upon which they specialize. An alternative hypothesis, that food cravings result from nutrient shortages 86, predicts the opposite: preference for novel foods rich in micronutrients that had been lacking in the previous simple diet.

### Cravings should be associated with lower parasympathetic (vagal) tone, and blocking the vagus nerve should reduce food cravings

If microbial control is mediated through the vagus nerve, then microbial signals should interfere to some extent with the physiological regulation coordinated by the vagus nerve. Vagal tone can be easily measured through respiratory sinus arrhythmia 87, the extent to which the heart rate changes in response to inspiration and exhalation. We predict that people experiencing cravings should have lower vagal tone. Furthermore, it is possible to block or sever the vagus, which we predict would subdue microbial signaling via the vagus nerve, and thereby alter food preferences. This would be consistent with studies showing that blocking the vagus nerve can lead to weight loss 49,50.

### Microbial diversity should affect food choices and satiety

Certain features of microbial ecology, such as population size, would be expected to influence a microbe's capacity to manipulate the host. Microbial communities with low alpha (intrasample) diversity might be more prone to overgrowth by one or more species, giving those organisms increased ability to manufacture behavior-altering neurochemicals and hormones. By comparison, in microbial communities with high alpha diversity any single microbial species will tend to occur at lower abundance. Highly diverse gut microbiotas tend to be more resistant to invasion by pathogenic species than less diverse microbiotas 88. In addition, a phylogenetically diverse community will likely contain competing groups whose influences may counteract each other. Furthermore, in a diverse microbial environment, microbes will likely expend resources on competing and cooperating (e.g. via cross-feeding), rather than on manipulating their host. Supporting the hypothesis that a more diverse microbiota causes fewer cravings, gastric bypass surgery has a twofold effect: increasing alpha diversity in the gut microbiota as well as reducing preference for high fat, high carbohydrate foods 89–91. Food preferences of germfree mice inoculated with low versus high diversity microbial communities could provide a test of this prediction. Similarly, probiotics that increase microbiota diversity in humans are predicted to reduce cravings more than control treatments that do not increase diversity.

### Excess energy delivery to the gut may reduce microbial diversity

Besides affecting cravings for specific nutrients, conflict between host and microbiota is expected to impact satiety and overall calorie consumption because optimal energy intake is likely to differ between the host and members of the gut microbiota. Excess energy delivered to the gut, beyond what is optimal for the host, might provide energy substrates for microbial growth, permitting certain species to bloom, potentially overwhelming inhibition by competitor organisms and the immune system. Energy excess is predicted to reduce diversity as a result, leading to a vicious cycle of reduced diversity, increased manipulation and chronic energy excess. Such a positive feedback mechanism could drive long-term changes in satiety, harming the host by causing obesity. Experimental increases in gut microbiota diversity are expected to change the satiety setpoint, favoring decreased food intake by the host 92.

### High gut diversity may inhibit density-dependent microbial manipulation

One explanation for the health benefits of intestinal diversity is the inhibition of quorum sensing microbes from achieving a quorum. Quorum sensing is a cell–cell communication system used by many gut bacteria to regulate density-dependent conditional strategies, including virulence factor expression and changes in growth. For instance, the common human commensal and pathogen *S. aureus* uses the accessory gene regulator system (AGR) of quorum sensing to regulate toxin and other virulence genes. When *S. aureus* reaches high density, AGR switches from expression of genes involved in colonization and attachment to those involved in tissue invasion 93. Quorum sensing may be one route that microbes can use to coordinate behavior in order to manipulate host eating behavior and enhance resource delivery. It is in the host's interest to prevent bacteria from reaching the threshold density for expression of virulence toxins and proteases. From a translational perspective, treatments that increase microbial diversity might prevent some microbe populations from reaching the density required for a quorum, thus limiting their capacity to manipulate host behavior.

### Interrogation of host and microbiota genomes should reveal a signaling arms race

There has been little work to study the co-evolution of the microbiome and their host genomes 11,94, and what there is has tended to focus on mutualism rather than evolutionary conflict between microbes and their hosts. We hypothesize that there has been a genomic arms race in which microbes have evolved genes to manipulate their hosts (particularly analogs of human signaling molecules such as neuropeptides and hormones) and corresponding host genes have evolved to prevent that manipulation where it conflicts with the host's fitness interests. Comparative genomic analyses may reveal such co-evolutionary patterns, and they have already identified adaptations specific to obligate commensal microbes 95,96.

### Food preferences may be contagious

One intriguing implication of microbially induced cravings is that preferences for certain foods may be contagious 97. Both the fecal and oral microbiota are more similar among cohabiting family members compared to non-cohabiting individuals 98. If the food preferences of one person in a household influence the food consumption of the household, any specialist gut microbes adapted to that diet would then tend to flourish in the other household members. Even worse, the obesity epidemic could be contagious as a result of obesity-causing microbes transmitted from person to person. A social network study of 12,067 people found that a person's chance of becoming obese increased by 57% if a friend had become obese 99. This raises up the possibility that cravings and associated obesity might not be *socially* contagious (e.g. through changes in norms) as the authors of the social network study suggest 99, but rather truly infectious, like a cold 75. This proposition could be tested by experimentally selecting for a microbiome that generates a particular food preference in animals, as above. As others have proposed, if food preferences are contagious, then co-housing those manipulated animals with germ-free animals should lead to transmission of food preference 7,100.

## Alternative hypotheses for unhealthy eating and obesity

There are a number of existing hypotheses for the prevalence of obesity and our cravings for unhealthy foods, including addiction/lack of willpower, environmental mismatch, and nutrient shortages. A microbial cause is not mutually exclusive of other alternatives such as nutrient deprivation. In this section, we review each of these alternative hypotheses. We find that none of these hypotheses is completely consistent with the data on cravings, food preferences, and obesity.

### Lack of willpower is not sufficient to explain unhealthy eating

Conventional wisdom often blames unhealthy eating on a lack of willpower. However, binge eating is not just a matter of mental control 101; food cravings are unlike other cravings. Many other addictions, such as drugs and alcohol, require ever-increasing doses to maintain the same mood-altering effect. This habituation does not happen with food. For some individuals, the more they indulge their food cravings, the more enjoyment they get from them 102. These results, and recent work showing distinct mechanisms of food-reward and morphine sensitization in mice suggest that overeating has a different underlying mechanism from drug abuse, and is not consistent with an addiction 103.

### Mismatch with scarce resources in our ancestral environment is not sufficient to explain unhealthy eating

Food preferences are thought to arise from a complex interaction between genes, environment, and culture. The modern food environment is vastly different from that of our evolutionary ancestors: the human ancestral diet is thought to contain foods far lower in salt, simple carbohydrates, and saturated fat than the typical Western diet 104. This discordance, or environmental mismatch, has been cited as the source of “diseases of civilization,” including obesity, cancer, and cardiovascular disease 105. Similar logic postulates that past scarcity of calorie dense foods and critical micronutrients has also shaped modern food preferences. The traditional diet of pre-agricultural humans relied on low-carbohydrate plant foods and game, low in fat. Among hunter gatherers, food acquisition efforts have been shown to prioritize energy dense foods, gathered in a pattern that maximizes energy capture relative to energy expenditure. This strategy, described as optimal foraging theory, is fitness enhancing in an environment where energy dense foods were rare and hard to acquire 106. Under this hypothesis, in the modern food environment with abundant food and sedentary lifestyles, once-adaptive physiologic mechanisms regulating energy intake and expenditure have gone awry, leading to overeating and obesity.

Despite the intuitive appeal of this hypothesis, a number of food preferences and cravings are not in accord with its predictions. For example, one of the most common modern cravings involves a food that ancient hominids never knew and which fulfills no nutritional requirement: chocolate 102. The hypothesis that environmental mismatch explains diseases caused by diet has also been criticized by others as overly simplistic 86.

### Nutrient deprivation is not sufficient to explain unhealthy eating

A similar hypothesis proposes that cravings result from nutrient shortage 84. For instance, fruit flies seek out specific nutrients after deprivation 107. However, this hypothesis does not explain many findings regarding cravings in humans. Food cravings strike even in times of plenty 108,109, and often foods that would satisfy a supposed nutrient shortage are not the ones that are craved 110. Furthermore, fasting reduces cravings 111–113 rather than increasing them, as would be expected from the nutrient shortage hypothesis. The same pattern holds for cravings of non-food items such as clay and earth 114. Young and colleagues subjected geophagy (earth-eating) to a systematic review and concluded that human geophagy is not driven by nutrient scarcity 114.

## Conclusions

Modern biology suggests that our bodies are composed of a diversity of organisms competing for nutritional resources. Evolutionary conflict between the host and microbiota may lead to cravings and cognitive conflict with regard to food choice. Exerting self-control over eating choices may be partly a matter of suppressing microbial signals that originate in the gut. Acquired tastes may be due to the acquisition of microbes that benefit from those foods. Our review suggests that one way to change eating behavior is by intervening in our microbiota.

It is encouraging that the microbiota can be changed by many interventions, hence facilitating translation to the clinic and public health efforts. Microbiota community structure changes drastically within 24 hours of changing diet 14,115 or administration of antibiotics 116. Fecal transplants have shown efficacy in treating a variety of diseases 117. The best approaches to managing our microbiota are still open questions. Many studies of the effects of gut microbes on health have focused on identifying individual taxa that are responsible for human diseases, an approach that has been largely unsuccessful in generating predictive hypotheses. Studies have identified conflicting different groups of microbes associated with various diseases, including obesity 118,119. In other domains, it has proven useful to shift the level of analysis from properties of the individual to properties of the population, e.g. diversity 120. Until we have a better understanding of the contributions and interactions between individual microbial taxa, it may be more effective to focus interventions on increasing microbial diversity in the gut.

Competition between genomes is likely to produce a variety of conflicts, and we propose that one important area, impacting human health, is in host eating behavior and nutrient acquisition. Genetic conflict between host and microbiota – selecting for microbes that manipulate host eating behavior – adds a new dimension to current viewpoints, e.g. host-microbiota mutualism 11, that can explain mechanisms involved in obesity and related diseases.
